# Spontaneous Heterotopic Triplet Pregnancies With Single Intrauterine and Twin Tubal Pregnancies Post‐Levonorgestrel Use: Case Report and Literature Review

**DOI:** 10.1002/ccr3.70080

**Published:** 2025-01-06

**Authors:** Azadeh Tarafdari, Nasim Eshraghi, Hawraa Shbeeb, Marzie Poorabdoli, Marjan Ghaemi, Mohammadamin Parsaei

**Affiliations:** ^1^ Department of Obstetrics and Gynecology, Imam Khomeini Hospital Complex, Vali‐e‐Asr Hospital Tehran University of Medical Sciences Tehran Iran; ^2^ Vali‐e‐Asr Reproductive Health Research Center Family Health Research Institute, Tehran University of Medical Sciences Tehran Iran; ^3^ Breastfeeding Research Center Family Health Research Institute, Tehran University of Medical Sciences Tehran Iran

**Keywords:** case report, ectopic pregnancy, emergency contraception, heterotopic pregnancy, triplet pregnancy

## Abstract

A rare spontaneous triplet heterotopic pregnancy occurred in a patient using emergency contraception. This highlights the need to consider heterotopic pregnancy in differential diagnoses for patients presenting with abdominal pain or vaginal bleeding, even with detected intrauterine pregnancies, especially after failed emergency contraception, necessitating thorough laboratory and ultrasonographic diagnostic work‐up.

## 1 Introduction

Heterotopic pregnancy, a rare condition characterized by the simultaneous presence of an intrauterine and an ectopic pregnancy, has become more common with the increased use of assisted reproductive technologies (ART) [1]. Factors such as transferring multiple embryos and various ovulation induction methods have contributed to this rise [2]. Conversely, heterotopic pregnancies resulting from spontaneous conception are less frequent and typically occur in individuals with known risk factors, including a history of pelvic inflammatory disease, previous ectopic pregnancy, or abdominal surgeries involving the fallopian tubes [3].

In this study, we present an exceptionally rare case of a triplet heterotopic pregnancy involving a single intrauterine pregnancy and twin monochorionic monoamniotic tubal pregnancies, achieved through spontaneous conception in a patient without known risk factors for heterotopic pregnancies. The findings of this case were reported strictly following the Clinical Case Reporting Guideline Development (CARE) guidelines [4].

## 2 Case History/Examination

A 29‐year‐old primigravid woman presented to the Infertility Clinic of the Vali‐e‐Asr Hospital in the Imam Khomeini Hospital Complex, affiliated with Tehran University of Medical Sciences, Tehran, Iran, with persistent vaginal spotting and abdominal pain over the past month, following an induced abortion. The patient reported having regular menstrual cycles, with no history of ARTs or the use of oral or injectable contraceptives. Additionally, she had not taken any other medications in the last 3 months leading up to the visit and had no history of prior abdominopelvic surgeries. The patient also reported no use of emergency contraceptive agents within the past year.

Approximately 2 months before her visit, the patient took two 0.75 mg oral levonorgestrel emergency contraceptive pills (Tansy 1.5 mg tablets, Atipharmed, Tehran, Iran) within 24 h after unprotected intercourse, administered during the mid‐cycle (ovulation phase) of her menstrual cycle. When her expected period did not occur, she underwent a qualitative beta‐human chorionic gonadotropin (β‐hCG) test approximately 1 month after conception, which was positive, indicating a spontaneous pregnancy and failure of the emergency contraception. Subsequent ultrasonography confirmed an intrauterine gestational sac, measuring 4 weeks and 3 days based on mean sac diameter and 5 weeks based on the last menstrual period (LMP). Unwilling to continue the pregnancy, the patient self‐administered four 200 mcg sublingual and four 200 mcg vaginal misoprostol tablets (Cytotec 200 mcg tablets, Pfizer, NY, USA) to induce abortion, resulting in the expulsion of the pregnancy tissue.

Despite the abortion, the patient continued to experience vaginal spotting and abdominal pain, prompting a visit to another center 20 days later. In the transvaginal sonography (TVS), a focal echogenic area measuring 41 × 14 mm^2^ in the endometrial cavity was detected, suggestive of residual placental tissue and pregnancy remnants. Additionally, the TVS revealed a focal cystic mass adjacent to the right ovary, indicative of a right tubal pregnancy.

**FIGURE 1:**
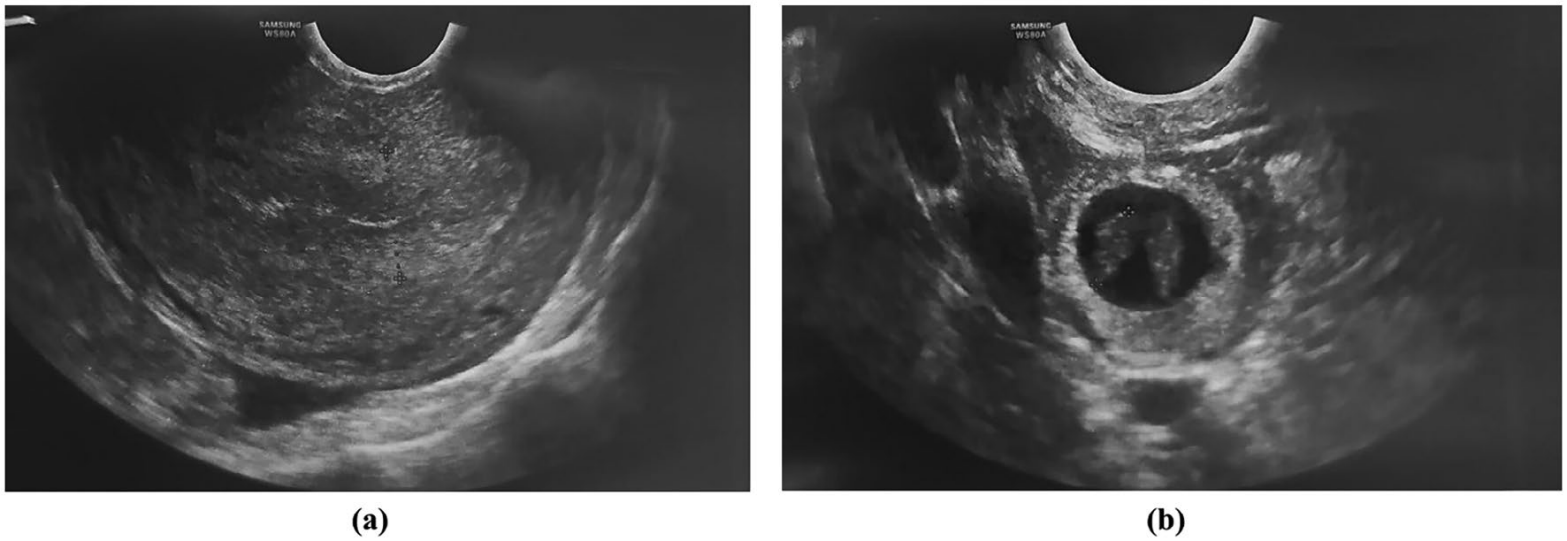
(a) The endometrium appears thick and echogenic, with a maximum thickness of 17 mm. No internal vascularity is observed within the endometrial content, which is suggestive of retained hemorrhage. There is no evidence of an intrauterine pregnancy. (b) A complex extra‐adnexal cyst with an echogenic wall is detected in the paraovarian region (separate from the ovary), measuring 28 × 20 mm^2^. This cyst exhibits the ‘ring of fire’ sign, indicating prominent peripheral vascular flow. The cyst contains two echogenic components, resembling fetal poles. The crown‐rump length (CRL) of 10 mm corresponds to a gestational age of approximately 7 weeks and 1 day, while the CRL of 8 mm corresponds to approximately 6 weeks and 6 days. No fetal cardiac activity is observed.

Follow‐up TVS with Doppler examination 4 days later revealed a thick, echogenic endometrium with a maximum thickness of 17 mm and no internal vascularity within the endometrial content, suggesting the presence of retained pregnancy products in the uterine cavity. Additionally, an extrauterine right adnexal cystic structure with an echogenic wall was identified. A complex extra‐adnexal cyst in the paraovarian region, measuring 28 × 20 mm^2^, displayed the ‘ring of fire’ sign with the prominent peripheral vascular flow and contained two echogenic components resembling fetal poles. The crown‐rump lengths (CRLs) of 10 and 8 mm corresponded to gestational ages of approximately 7 weeks and 1 day, and 6 weeks and 6 days, respectively, indicating a monochorionic monoamniotic tubal pregnancy, though no fetal cardiac activity was detected. Mild free fluid in the pelvic cavity was noted, with no signs of tubal rupture or hemoperitoneum (Figure 1).

At the presentation in our center, the patient was 8 weeks and 3 days pregnant based on her LMP. Physical examination revealed no abdominopelvic tenderness, rebound tenderness, or palpable masses. Mild vaginal bleeding and a dilated cervix were noted, suggestive of an incomplete abortion. No vaginal discharge was observed. Her vital signs were stable: blood pressure 120/70 mmHg, pulse rate 97 bpm, respiratory rate 20 breaths/min, temperature 36.5°C, and oxygen saturation 96%. Also, her β‐hCG level was 98,000 mIU/L.

## 3 Methods

Considering the presence of a twin tubal pregnancy and the high risk of rupture, and after explaining the rationale, the patient was informed about the necessity of undergoing surgical treatment. Informed verbal and written consent were acquired from the patient in this regard. Subsequently, the patient was transferred to the operating room. Initially, the residual intrauterine placenta was removed via hysteroscopy. Then, a laparoscopic right salpingectomy was performed due to the large size of the fetuses, without complication.

## 4 Conclusions and Results

The patient was discharged one‐day post‐surgery in good clinical condition with stable vital signs and no symptoms. Additionally, two specimens were sent for pathological examination: endometrial tissue obtained via hysteroscopy and the right fallopian tube obtained from laparoscopic salpingectomy. The endometrial specimen consisted of gray tissue with blood clots, suggestive of decidualized endometrium, and contained microvillous structures and products of conception, indicative of an intrauterine pregnancy that likely resulted in abortion. The right fallopian tube specimen comprised a tubular, tan tissue measuring 8 cm in length and 2 cm in diameter, with villus‐like structures and areas of hemorrhage, along with a separate 2 × 2 × 1 cm^3^ piece suggestive of a product of conception.

## 5 Discussion

Heterotopic pregnancy is a rare condition characterized by the simultaneous existence of intrauterine and extrauterine pregnancies [5]. Typically, the extrauterine fetus is located in the fallopian tube, but other locations such as the cornua, cesarean scar, and cervix have also been reported [6]. Historically, the incidence of heterotopic pregnancy was 1 in 30,000 pregnancies, but recent data indicates an increase to 1.5 in 1000 pregnancies [7]. This rise is largely attributed to the higher incidence of pelvic inflammatory disease and the increasing use of ART for infertility treatment [8, 9]. While most heterotopic pregnancies develop following ART, particularly after ovulation induction or embryo transfer, there have also been numerous cases following spontaneous conception [6]. Whereas ART remains the primary risk factor, increasing the incidence by 90 times [10], other risk factors include previous ectopic pregnancies, prior tubal surgeries, documented tubal pathologies, and pelvic infections [9, 11].

Since the first documented case in 1903 from a natural cycle pregnancy [12], most reported triplet heterotopic pregnancies have resulted from ART [13, 14, 15]. However, our review of the literature identified a total of 24 other cases of spontaneously conceived triplet heterotopic pregnancies (Table 1). Of them, 20 studies reported the presence of a twin intrauterine pregnancy and a single extrauterine pregnancy [12, 16, 17, 19, 20, 23, 24, 25, 26, 27, 28, 29, 30, 31, 32, 33, 34, 36, 37, 38], and four studies reported cases of single intrauterine pregnancies accompanied by twin extrauterine pregnancies [18, 21, 22, 35]. Except for two cases initially treated with systemic methotrexate due to the patients' unwillingness to preserve the intrauterine pregnancies [20, 32], one of which subsequently failed and required an open hysterectomy procedure [20], all other 22 cases were managed surgically as first‐line treatment for the removal of the ectopic pregnancy. Eleven patients underwent open surgeries [12, 16, 17, 18, 21, 22, 23, 28, 30, 33, 34, 37], with one also undergoing suction evacuation of the intrauterine pregnancy [22]. Additionally, nine patients underwent laparoscopic intervention [19, 24, 25, 26, 27, 29, 35, 36, 38], one of whom also underwent suction evacuation of the intrauterine pregnancy [36]. One case underwent only soft curettage, aspiration, foley catheter placement, and cerclage, specifically for evacuation of the single cervical pregnancy [31].

**TABLE 1:** Characteristics of all reported cases of spontaneous heterotopic triplet pregnancies in the literature.

Study	Country	Age; year	G/P	GA; w	EP history	Contraception	Symptoms	Type of pregnancy	Ultrasonographic findings	Tubal rupture	Management	Outcome
Marshall (1903) [12]	United Kingdom	30	5/4	12	No	No	Abdominal pain, pallor, cold sweat, vomiting, thirst, restlessness, tachycardia, and tachypnea.	Twin intrauterine + Single extrauterine (tubal)	NA	Yes	Open hysterectomy + bilateral salpingectomy	The patient died 2 days post‐op due to massive blood loss.
Nguyen‐Tran and Troy (2000) [16]	United States	NA	NA	NA	NA	NA	NA	Twin intrauterine + Single extrauterine (tubal)	Laterouterine gestational sac with heart activity	NA	Open salpingectomy	The patient delivered healthy twins at term.
Anziekoro (2002) [17]	Nigeria	24	1/0	8	No	NA	Abdominal pain, weakness, pallor, and tachycardia	Twin intrauterine + Single extrauterine (tubal)	NA	Yes	Open salpingectomy	The patient delivered healthy twins at term.
Alsunaidi (2005) [18]	Saudi Arabia	42	6/3	8	No	No	Abdominal pain	Single intrauterine + Twin extrauterine (tubal)	Hemoperitoneum, laterouterine sac with 2 yolk sacs	Yes	Open salpingectomy	The patient delivered a healthy neonate at term.
Cholkeri‐Singh and Labarge (2007) [19]	United States	30	4/1	9	No	No	Abdominal pain, nausea, and vomiting	Twin intrauterine + Single extrauterine (tubal)	Hemoperitoneum, ovary, not viewed	Yes	Laparoscopic salpingostomy (+ hydrodissection)	The patient delivered healthy preterm twins (GA = 34 w) via C/S.
Matijević et al. (2008) [20]	Croatia	37	5/2	8	No	No	Heavy vaginal bleeding	Twin intrauterine + Single extrauterine (cervical)	2 laterouterine gestational sacs with heart activity and one live embryo in the cervical canal	No	Systemic methotrexate + open hysterectomy	The patient was discharged 6 days post‐op in a stable condition.
Simsek et al. (2008) [21]	Turkey	37	2/0	9	No	No	Abdominal pain, nausea, and vomiting	Single intrauterine + Twin extrauterine (tubal)	Hemoperitoneum, 1 laterouterine gestational sac with heart activity and a twin tubal pregnancy without heart activity	Yes	Open salpingectomy	The patient delivered a healthy neonate at term.
Dahiya et al. (2011) [22]	India	30	2/1	7	No	No	Abdominal pain, pallor, and tachycardia	Single intrauterine + Twin extrauterine (Bilateral fallopian tubes)	Hemoperitoneum	Yes (left)	Open salpingostomy (left) and salpingectomy (right) + suction evacuation	The patient was discharged 4 days post‐op in a stable condition.
Rimawi et al. (2013) [23]	United States	34	2/1	9	Yes	Yes	Abdominal pain and vaginal bleeding	Twin intrauterine + Single extrauterine (tubal)	NA	Yes	Open salpingectomy	The patient delivered healthy preterm twins (GA = 30 w) via C/S.
Arsala and Danso (2014) [24]	Australia	27	5/2	4	No	No	Abdominal pain	Twin intrauterine + Single extrauterine (tubal)	Hemoperitoneum, 2 intrauterine, and 1 right adnexal gestational sac, all with cardiac activity	Yes	Laparoscopic salpingectomy	At day 8 post‐op, a single continuing viable intrauterine pregnancy and a miscarriage of the the second twin was observed.
Nnoli et al. (2015) [25]	United States	29	2/1	8	No	Yes	Abdominal pain and nausea	Twin intrauterine + Single extrauterine (tubal)	Twin gestation with an ectopic pregnancy situated in the right tube, close to the right ovary	Yes	Laparoscopic fimbriectomy	Her twin intrauterine pregnancy remained uncomplicated until 24‐week follow‐up examinations.
Kotylar et al. (2016) [26]	United States	35	4/2	15	NA	NA	Abdominal pain	Twin intrauterine + Single extrauterine (tubal)	A mixed echogenicity mass adjacent to the right ovary with a small amount of free fluid.	No	Laparoscopic salpingectomy	Intrauterine pregnancies were viably continued, but the fetal demise of both twins was noted at the GA of 22 weeks.
Rengaraj et al. (2016) [27]	India	26	3/2	8	No	NA	Mild pallor	Twin intrauterine + Single extrauterine (tubal)	Live twin intrauterine gestation with complex mass in right adnexa (tubal abortion) with minimal free fluid	No	Laparoscopic salpingostomy	The patient delivered a healthy neonate at term.
Ojo et al. (2017) [28]	Nigeria	40	4/2	10	No	NA	Abdominal pain, vaginal bleeding, and hypotension	Twin intrauterine + Single extrauterine (cornual)	Hemoperitoneum, 2 fetuses in the fundal regions (one with cardiac activity)	Yes	Open cornual resection	The patient delivered the viable fetus from the twin intrauterine pregnancy at term via C/S.
Torky and Elaa (2017) [29]	Egypt	29	4/0	10	NA	NA	Abdominal pain	Twin intrauterine + Single extrauterine (tubal)	Viable intrauterine twins,2 unilocular simple ovarian cysts, and right adnexal mass inseparable from the ovary	No	Laparoscopic salpingostomy	The patient delivered healthy preterm twins (GA = 34 w) via spontaneous vaginal delivery.
Guimarães et al. (2019) [30]	Brazil	21	1/0	8	NA	NA	Abdominal pain	Twin intrauterine + Single extrauterine (tubal)	A 2‐cm tubular structure in the right iliac fossa was reported as acute appendicitis and an 8‐week viable intrauterine twin gestation	Yes	Open salpingectomy	The patient delivered healthy preterm twins (GA = 36 w) via C/S.
Socha and Wolski (2019) [31]	Poland	27	1/0	7	NA	NA	Vaginal bleeding	Twin intrauterine + Single extrauterine (cervical)	Triplet intrauterine pregnancy with 2 live embryos and 1 viable intracervical embryo	No	Soft curettage and aspiration + Foley catheter placement + Shirodkar cerclage	The patient delivered healthy preterm twins (GA = 32 w).
Yelurkar et al. (2019) [32]	India	32	3/1	8	No	NA	Abdominal pain and vaginal bleeding	Twin intrauterine + Single extrauterine (cesarean scar)	2 intrauterine and 1 cesarean scar gestational sacs	No	Systemic methotrexate and leucovorin	8 weeks following the methotrexate administration, all 3 gestational sacs shrank.
Nkurunziza et al. (2020) [33]	Rwanda	34	6/3	18	NA	Yes	Abdominal pain, weakness, tachycardia, and tachypnea	Twin intrauterine + Single extrauterine (cornual)	Intrauterine twin pregnancy with no cardiac activity for both fetuses. A third fetus was seen outside the uterus without cardiac activity and there was free fluid in the abdomen.	Yes	Open subtotal hysterectomy	The patient was discharged 4 days post‐op in a stable condition.
Ntounis et al. (2021) [34]	Greece	43	4/3	7	NA	NA	Abdominal pain and vaginal bleeding	Twin intrauterine + Single extrauterine (tubal)	Hemoperitoneum, 2 intrauterine pregnancies with fetal heart activities	Yes	Open salpingectomy + oophorectomy	The patient delivered the viable fetus from the twin intrauterine pregnancy at preterm (GA = 36 w) via C/S.
LaPorte et al. (2022) [35]	United States	29	3/2	7	No	NA	Abdominal pain	Single intrauterine + Twin extrauterine (cornual)	Hemoperitoneum, a fetal pole measuring 8 weeks with cardiac activity surrounded by a sizable layer of myometrium, as well as an additional fetal pole with cardiac activity laterally with a thin surrounding layer of myometrium	Yes	Laparoscopic salpingectomy + cornuectomy	Laparoscopic excision of 2 EPs was safely performed in this patient with maintenance of a viable, intrauterine pregnancy.
Aldrich et al. (2023) [36]	United States	30	6/4	6	No	NA	Pelvic pain and vaginal bleeding	Twin intrauterine + Single extrauterine (cesarean scar)	2 intrauterine pregnancies (1 with cardiac activity) and 1 live pregnancy at the cesarean scar	No	Laparoscopic salpingectomy (bilateral) + suction dilation and curettage	The patient was seen for a follow‐up 2 weeks postoperatively, at which time she reported feeling well and had no complications.
Neola et al. (2023) [37]	Italy	35	3/2	12	No	NA	Pelvic pain and fainting	Twin intrauterine + Single extrauterine (tubal)	Hemoperitoneum, monochorionic diamniotic twin pregnancy in the uterus with cardiac activity present for both fetuses and an anechoic area surrounded by a hyperechoic layer with a vascular ring in the right adnexa.	Yes	Open salpingectomy	Her twin intrauterine pregnancy remained uncomplicated until 20‐week follow‐up examinations.
Kassi et al. (2024) [38]	United States	34	2/0	7	No	NA	Abdominal pain and nausea	Twin intrauterine + Single extrauterine (tubal)	Hemoperitoneum, a twin monochorionic diamniotic intrauterine pregnancy with heart activity and an echogenic ring‐like structure with possible embryonic structures noted within the left adnexa, with the peripheral flow and no cardiac activity	Yes	Laparoscopic salpingectomy	The patient delivered the viable fetus from the twin intrauterine pregnancy at preterm (GA = 32 w) via C/S.

Abbreviations: C/S, cesarean section; EP, ectopic pregnancy; G, gravidity; GA, gestational age; NA, not available; P, parity; w, weeks; y, years.

In terms of the treatment outcomes, except for one case that resulted in mortality due to massive hemorrhage [12], in most cases where there was intent to continue the intrauterine pregnancy, this goal was achieved [16, 17, 18, 19, 21, 23, 25, 27, 28, 29, 30, 31, 34, 35, 37, 38]. However, in one case, both twins were subsequently diagnosed as demised [26], while in another case, one of the twins was diagnosed as demised [24]. Conversely, in cases where the termination of intrauterine pregnancy was also intended, all of the pregnancies were successfully terminated without significant adverse events for the patients [20, 22, 32, 33, 36]. These findings suggest that although rare, spontaneous triplet heterotopic pregnancies can occur, with optimal outcomes when detected and managed promptly.

Our case is unique in that it occurred following the failed use of emergency levonorgestrel pills. This suggests that the failure of emergency conception may also be a potential risk factor for developing ectopic and heterotopic pregnancies. Previous research has indicated that while emergency contraception does not necessarily increase the overall incidence of ectopic pregnancies [39], it does highlight a significant rate of ectopic pregnancy in cases of failed treatment [40, 41, 42]. The literature identifies three prominent mechanisms of action for levonorgestrel: delaying or preventing ovulation, slowing egg migration due to impaired ciliary function, and inhibiting sperm function and migration. It is theorized that emergency contraceptive pills fail when ovulation has already occurred before the medication is taken. In such cases, while the ovum is in the fallopian tube, the impaired ciliary function caused by emergency contraceptive pills may facilitate an ectopic pregnancy. Since emergency contraceptive pills cannot prevent the growth of a fertilized ovum, an ectopic pregnancy may proceed undetected [40, 43, 44].

Surprisingly, our case is the first in the literature to report the co‐occurrence of a twin ectopic pregnancy alongside an intrauterine pregnancy following the use of emergency contraception pills, making it unique. This suggests that even when an intrauterine pregnancy is detected after the failed use of emergency contraception, clinicians should consider the potential existence of extrauterine pregnancies during their initial assessments and a comprehensive abdominopelvic ultrasonographic examination is necessary to rule out the existence of extrauterine pregnancies. Such vigilance is crucial to prevent missed diagnoses, as in our case, which can pose a significant risk to the patient's life.

Furthermore, diagnosing heterotopic pregnancies poses significant challenges, as the presence of an intrauterine pregnancy can obscure the detection of an extrauterine one. Consequently, extrauterine pregnancy is often discovered at more advanced stages, frequently after signs of tubal rupture, necessitating prompt surgical intervention [45]. This issue can be further complicated in cases of triplet heterotopic pregnancies, where the risk of tubal rupture is doubled in twin tubal gestations and the risk of missing the extrauterine pregnancy is higher in cases of detected twin intrauterine pregnancies, potentially allowing the extrauterine pregnancy to progress to more advanced stages and lead to tubal rupture and other serious complications.

This point was also presented in the findings of our review, suggesting the presence of tubal rupture in 80% (16/20) of cases at the time of detection of heterotopic triplet pregnancies. Therefore, in patients with previously detected intrauterine pregnancies, even twin pregnancies, who develop sudden or gradually increasing abdominal pain or vaginal bleeding, the possibility of an extrauterine pregnancy should be considered. This is particularly crucial in pregnant women presenting with signs of unstable hemodynamics or acute abdomen, where a ruptured tube must be considered. By doing so, prompt surgical management can then be employed to save the mother's life and maintain the intrauterine pregnancy.

Also, it is important to highlight a key differential diagnosis for spontaneous heterotopic pregnancy in women previously diagnosed with an intrauterine pregnancy who present with symptoms such as abdominal pain or vaginal bleeding. This differential includes the concurrent existence of a complete hydatidiform mole alongside a normal fetus, which can be misinterpreted as a heterotopic pregnancy. Given the prevalence of late diagnoses of hydatidiform moles in cases with prior intrauterine pregnancies, it is crucial to consider this possibility and conduct a thorough evaluation, particularly with ultrasonography, in pregnant women exhibiting these symptoms to avoid severe complications arising from delayed diagnosis [46].

Furthermore, the reviewed literature indicates that systemic methotrexate treatment for heterotopic pregnancies may yield suboptimal outcomes in patients who do not wish to preserve their intrauterine pregnancies [20]. In such cases, surgical management should be generally preferred as a first‐line treatment. Moreover, when the goal is to maintain the intrauterine pregnancy while removing the extrauterine pregnancies, salpingectomy should be favored over salpingostomy. Although three studies have reported successful management and live birth outcomes following laparoscopic salpingostomy [22, 27, 29], we recommend salpingectomy due to the difficulty in monitoring postoperative β‐hCG levels to rule out persistent ectopic pregnancies, which pose significant risks and are more common after salpingostomy compared to salpingectomy [47].

Collectively, our case and literature review highlight that, although extremely rare, heterotopic triplet pregnancies can occur after spontaneous conception. This underscores the need to consider an extrauterine pregnancy as a differential diagnosis in patients with detected intrauterine pregnancies when they present with abdominal pain or vaginal bleeding, even when a twin intrauterine pregnancy is confirmed. Our case also suggests that failed emergency contraception might increase the risk of ectopic pregnancies and could be a potential risk factor for developing ectopic or heterotopic pregnancies. Therefore, it is crucial to perform a thorough abdominopelvic ultrasound in suspected cases to rule out any extrauterine pregnancies.

## Author Contributions

**Azadeh Tarafdari:** conceptualization, data curation, investigation, project administration, resources, supervision, validation, writing – review and editing. **Nasim Eshraghi:** data curation, validation, writing – original draft. **Hawraa Shbeeb:** investigation, resources, writing – original draft. **Marzie Poorabdoli:** investigation, validation, writing – review and editing. **Marjan Ghaemi:** investigation, methodology, resources, writing – original draft. **Mohammadamin Parsaei:** investigation, methodology, resources, software, validation, visualization, writing – original draft.

## Ethics Statement

We strictly adhered to the principles of the Declaration of Helsinki throughout the whole study process. After providing a thorough explanation of the necessity of the therapeutic procedure and its potential adverse events, informed verbal and written consent was obtained from the patient prior to the procedure.

## Consent

Written and formal consent for the publication of this case report was obtained from the patient.

## Conflicts of Interest

The authors declare no conflicts of interest.

## Data Availability

Data sharing is not applicable to this article as no datasets were developed or analyzed during the study.
